# Increased serum kallistatin levels in type 1 diabetes patients with vascular complications

**DOI:** 10.1186/2040-2384-2-19

**Published:** 2010-09-22

**Authors:** Alicia J Jenkins, Jeffrey D McBride, Andrzej S Januszewski, Connie S Karschimkus, Bin Zhang, David N O'Neal, Craig L Nelson, Jasmine S Chung, C Alex Harper, Timothy J Lyons, Jian-Xing Ma

**Affiliations:** 1 University of Melbourne, Department of Medicine, St Vincent's Hospital, Melbourne, Australia; 2 Harold Hamm Oklahoma Diabetes Center and Section of Endocrinology and Diabetes, Oklahoma University Health Sciences Center, Oklahoma City, OK, USA; 3 Department of Cell Biology, Oklahoma University Health Sciences Center, Oklahoma City, OK, USA; 4 University of Melbourne, Department of Ophthalmology, Melbourne, Australia

## Abstract

**Background:**

Kallistatin, a serpin widely produced throughout the body, has vasodilatory, anti-angiogenic, anti-oxidant, and anti-inflammatory effects. Effects of diabetes and its vascular complications on serum kallistatin levels are unknown.

**Methods:**

Serum kallistatin was quantified by ELISA in a cross-sectional study of 116 Type 1 diabetic patients (including 50 with and 66 without complications) and 29 non-diabetic controls, and related to clinical status and measures of oxidative stress and inflammation.

**Results:**

Kallistatin levels (mean(SD)) were increased in diabetic vs. control subjects (12.6(4.2) vs. 10.3(2.8) μg/ml, p = 0.007), and differed between diabetic patients with complications (13.4(4.9) μg/ml), complication-free patients (12.1(3.7) μg/ml), and controls; ANOVA, p = 0.007. Levels were higher in diabetic patients with complications vs. controls, p = 0.01, but did not differ between complication-free diabetic patients and controls, p > 0.05. On univariate analyses, in diabetes, kallistatin correlated with renal dysfunction (cystatin C, r = 0.28, p = 0.004; urinary albumin/creatinine, r = 0.34, p = 0.001; serum creatinine, r = 0.23, p = 0.01; serum urea, r = 0.33, p = 0.001; GFR, r = -0.25, p = 0.009), total cholesterol (r = 0.28, p = 0.004); LDL-cholesterol (r = 0.21, p = 0.03); gamma-glutamyltransferase (GGT) (r = 0.27, p = 0.04), and small artery elasticity, r = -0.23, p = 0.02, but not with HbA1c, other lipids, oxidative stress or inflammation. In diabetes, geometric mean (95%CI) kallistatin levels adjusted for covariates, including renal dysfunction, were higher in those with vs. without hypertension (13.6 (12.3-14.9) vs. 11.8 (10.5-13.0) μg/ml, p = 0.03). Statistically independent determinants of kallistatin levels in diabetes were age, serum urea, total cholesterol, SAE and GGT, adjusted r^2 ^= 0.24, p < 0.00001.

**Conclusions:**

Serum kallistatin levels are increased in Type 1 diabetic patients with microvascular complications and with hypertension, and correlate with renal and vascular dysfunction.

## Introduction

In diabetes, angiogenesis is disturbed, contributing to proliferative retinopathy, nephropathy, neuropathy, atherosclerosis, and impaired wound healing [1-6]. Hyperglycemia, hypertension, dyslipidemia, smoking, adiposity, inflammation and oxidative stress may promote vascular complications [1], and some effects of these stresses may be mediated by disturbances in the levels of or balance of pro- and anti-angiogenic factors, such as (anti-angiogenic) kallistatin.

Kallistatin, a tissue-kallikrein selective 427 amino acid 58-60 kD glycoprotein serpin has independent effects as a vasodilator and modulator of vascular growth, and anti-angiogenic, anti-oxidant and anti-inflammatory effects [7-13]. Found in a wide range of human tissues and fluids, including kidney, myocardium, blood vessels, plasma, and urine, [14,15], its levels are relevant to diabetes, a condition in which angiogenesis is disturbed and retinal, renal and cardiovascular damage is increased.

Kallistatin may predict and modulate diabetic angiopathy [16,17] and has potential for use as a therapeutic agent or target [18]. Clinical studies of circulating kallistatin levels are lacking. We hypothesize that, relative to healthy subjects, kallistatin levels may be increased in people with Type 1 diabetes or its microvascular complications, as a compensatory mechanism, and may be positively related to levels of retinal, renal and vascular damage, oxidative stress, inflammation and glycemia. We undertook a cross-sectional study of serum kallistatin levels in well-characterized Type 1 diabetic patients (with and without vascular complications) and in healthy controls, and related kallistatin levels to blood pressure, vascular function, microvascular complications, and traditional and novel vascular risk factors.

## Materials and methods

### Subjects and samples

The study, which conforms to the Declaration of Helsinki, was approved by the local Ethics Committee and each subject gave written informed consent. Patients were recruited from St Vincent's Hospital clinics, and controls were recruited from the community. Exclusion criteria were: end-stage renal disease (ESRD), cardiac arrhythmia, inflammatory conditions, recent (< 3 months) stroke, myocardial infarction, surgery (including laser therapy), infective illness, or diabetic ketoacidosis and anti-oxidant vitamin supplement intake. History and examination were performed, and complication status confirmed by treating clinicians. Retinopathy was defined as pre-proliferative or proliferative retinopathy requiring pan-retinal laser treatment. Nephropathy was defined as albuminuria (> 20 μg/minute) in repeated timed (12 or 24 hour) urine collections in absence of infection. Even if albuminuria subsequently regressed to normal range with treatment, subjects were still categorized as having nephropathy if they met these criteria. Cardiovascular disease (CVD) was defined as a documented myocardial infarction or angina with ECG changes and/or positive cardiac imaging study, a TIA or stroke, amputation, angioplasty, or vascular bypass surgery. Fasted subjects were evaluated pre-medication. Pulse wave analysis, including large and small artery elasticity (LAE and SAE), which correlate with pulse wave velocity and brachial artery flow mediated dilation respectively [19], was performed on rested supine subjects (Pulse Wave™ CR-2000, Hypertension Diagnostics Inc., Eagan, MN, USA), as previously [19-23]. Inter-measurement CVs for LAE and SAE were 7% and 5% respectively. St. Vincent's Clinical Chemistry measured HbA_1c_, full blood exam and ESR, serum lipids, renal and liver function, and a mid-stream urine for cell count, albumin/creatinine ratio and culture. Glomerular Filtration Rate (GFR) was calculated by the Cockgroft-Gault equation [24]. Research laboratory blood samples were centrifuged (3000 rpm, 10 min, 4°C) and aliquots stored (-86°C) until analysis.

*Kallistatin *levels were quantified by ELISA (R&D Systems, Inc. Minneapolis, MN). Ninety-six well microplates were coated with 100 μL/well of mouse anti-human Serpin A4 capture antibody in PBS (2.0 μg/mL), sealed and incubated overnight at room temperature (RT). Each well was aspirated and washed (0.05% Tween^® ^20 in PBS, pH 7.2-7.4) thrice during each washing step. Nonspecific binding to capture antibody was minimized by addition of 300 μL 1% filtered BSA in PBS (1 hour, RT). Recombinant human kallistatin standards were diluted to provide a seven point standard curve up to 8000 pg/mL. Sera were diluted (1/20000) in 1% BSA in PBS. After washing, 100 μL of each sample and standards were added to wells (in triplicate), plates sealed and incubated (2 hours, RT). After washing, 100 μL biotinylated goat anti-human kallistatin detection antibody in 1% BSA in PBS (200 ng/mL) were added to each well, sealed and incubated (2 hours, RT). After washing, 100 μL of streptavidin conjugated to horseradish-peroxidase (R&D Systems) were added per well, sealed and incubated (20 minutes, RT, not in direct light). After washing, 100 μL 1:1 H_2_O_2 _: tetramethylbenzidine was added to each well, sealed and incubated (20 minutes RT, not in direct light). Next, 50 μL 2N H_2_SO_4 _was added to stop the reaction. Absorbance (450 nm) (VICTOR3 V™ Multilabel Counter, PerkinElmer Life And Analytical Sciences, Inc, Waltham, Massachusetts) was used with best-fit equations for each standard curve (range 0-8000 pg/mL) to determine the kallistatin concentration. Intra and inter-assay CVs were ≤ 2% and < 11% respectively.

### Inflammation

To complement white cell counts (WCC) and ESR, *CRP *was measured by high-sensitivity nephelometry (Dade Behring, Marburg, Germany) [20-23]. *Soluble Cell Adhesion Molecules: *vascular cell adhesion molecule-1 (sVCAM-1), intercellular adhesion molecule-1 (sICAM-1) and endothelial leukocyte adhesion molecule-1 (sE-Selectin) were measured by ELISA (R&D Systems, Minneapolis, MN, USA) [20-23], with intra-and inter-assay CVs < 9%.

*Serum Cystatin C *was assayed by nephelometry (Dade-Behring, Marburg, Germany). Intra-assay and inter-assay CVs were 1.8 and 4.7%.

*Oxidized (Ox) LDL *was measured by ELISA (Mercodia, Uppsala, Sweden) with CVs < 10% and results expressed as OxLDL/LDL [23].

### Statistics

One-way ANOVA for continuous variables was used with a Tukey honest significant difference (HSD) post-hoc test for differences between groups when ANOVA P-value was < 0.05. Comparisons of prevalence of categorical variables used Chi-square test. Correlations were tested using Pearson correlation and Spearman rank coefficients. Non-normally distributed variables (Cystatin C, serum creatinine, urea, GFR, urinary albumin/creatinine ratio, triglycerides, CRP, ESR, WCC and Ox-LDL/LDL) were log_10 _transformed and results were expressed as geometric means (95% confidence intervals). To determine independent predictors of kallistatin levels age, gender, diabetes, and other significant correlates with kallistatin in bivariate analyses were tested in forward stepwise linear regression analysis. Renal function and inflammation indicators were examined sequentially and only those giving the strongest model (highest R-square value) were included in the final analysis. Variables were included in the final model if p < 0.30, tolerance > 0.1 and R^2 ^change in multiple regression > 0.03. Statistical significance was taken at p < 0.05. These subject numbers have at least 80% power to detect differences between means of kallistatin in diabetic patients (including comparisons of those with (DMCx) and without (DMNoCx) complications) and controls with a significance level (alpha) of 0.05.

## Results

*Subject characteristics *are shown in Table 1. Age, gender, and lipid profiles matched between diabetic and non-diabetic groups. Fasting glucose and HbA1c levels were higher in diabetes, but did not differ by diabetes complication status. Diabetes duration (range 0.1-63 years) and BMI were higher and renal function worse in those with complications (DMCx) than in non-diabetic subjects. Only six DMCx subjects had nephropathy without proliferative retinopathy. Nine diabetic subjects had macrovascular disease, which in each case was associated with microvascular complications. Complication-free diabetic subjects (DMNoCx) had no clinically evident micro- or macrovascular complications. BMI and renal function did not differ significantly between control and DMNoCx subjects. Systolic blood pressure and pulse pressure were increased and SAE was decreased in DMCx vs. controls, and these measures were lower in DMNoCx vs. DMCx, but did not differ from controls. Smoking was most prevalent in the DMNoCx group. Use of aspirin and drugs to treat hypertension and dyslipidemia was greatest in the DMCx group.

**Table 1:** Clinical and biochemical characteristics of healthy control subjects and Type 1 diabetic patients with microvascular complications (DMCx) and without complications (DMNoCx).

	Control (CON) n = 29	DMNoCx n = 66	DMCx n = 50	ALL DM n = 116	*P* All DM vs CON
Male gender,%	52	47	40	44	
Age, years	41 (14)	38 (14)	41 (14)	39 (14)	0.56
Diabetes duration, years	0	18 (12)^b^	27 (13)	22 (13)	
BMI, kg/m^2^	23.6 (2.9)	24.9 (3.4)^b^	27.6 (4.9)^a^	26.0 (4.2)	**0.004**
HbA1c, %	5.1 (0.5)	8.1 (1.3)^a^	8.4 (1.2)^a^	8.2 (1.3)	**< 0.001**
Glucose	4.8 (0.4)	12.9 (5.4)^a^	13.2 (5.8)^a^	12.9 (5.5)	**< 0.001**
***Vascular health measures***					
Systolic BP, mmHg	118 (12)	125 (12)^b^	136 (15)^a^	130 (15)	**< 0.001**
Diastolic BP, mmHg	69 (10)	70 (9)^b^	75 (10)	72 (10)	0.16
Pulse pressure, mmHg	49 (8)	55 (8)^a, b^	61 (10)^a^	57 (9)	**< 0.001**
Large artery elasticity, mL/mmHg × 10	18.1 (5.1)	16.7 (5.6)	15.7 (5.0)	16.2 (5.3)	0.082
Small artery elasticity, mL/mmHg × 100	7.7 (2.8)	6.7 (3.0)^b^	5.1 (2.7)^a^	6.0 (3.0)	**0.007**
***Renal function measures***					
Serum creatinine, mmol/L	0.08 (0.08-0.09)	0.08 (0.08-0.09)^b^	0.10 (0.09-0.11)^a^	0.09 (0.09-0.1)	0.06
Serum urea, mmol/L	5.4 (5.0-5.9)	5.2 (4.9-5.6)^b^	7.7 (6.6-9.0)^a^	6.1 (5.7-6.7)	0.15
Serum Cystatin C, mg/L	0.81 (0.76-0.86)	0.75 (0.72-0.78)^b^	0.97 (0.83-1.14)	0.83 (0.78-0.9)	0.76
GFR, ml/s/1.73 m^2^	92 (85-100)	99 (93-106)^b^	83 (74-95)	92 (87-98)	0.95
Urinary albumin/creatinine ratio mg/mmol	0.50 (0.32-0.77)	0.73 (0.60-0.89)^b^	5.41 (2.87-10.18)^a^	1.75 (1.24-2.47)	**0.003**
% on BP drugs	0	18^a, b^	70^a^	41	**< 0.001**
***Lipid profiles and medications***					
Total cholesterol, mmol/L	5.0 (0.9)	4.8 (1.0)^a^	5.4 (0.9)	5.1 (1.0)	0.67
Triglycerides, mmol/L	0.79 (0.64-0.98)	0.88 (0.78-1.00)	1.12 (0.95-1.32)^a^	0.98 (0.89-1.08)	0.06
LDL-cholesterol, mmol/L	3.0 (0.8)	2.9 (1.0)	3.1 (0.9)	3.0 (1.0)	0.93
HDL-cholesterol, mmol/L	1.6 (0.4)	1.5 (0.5)	1.6 (0.5)	1.6 (0.5)	0.66
% on lipid drugs	0.4	13	36^a^	23	**0.01**
***Inflammation and oxidative stress***					
White Cell Count, cellsx10^9^/L	5.6 (5.2-6.1)	6.2 (5.8-6.7)	7.0 (6.3-7.7)^a^	6.5 (6.2-6.9)	**0.02**
ESR, mm/h	4.8 (3.7-6.3)	7.0 (5.7-8.6)^b^	10.9 (8.5-14.3)^a^	8.4 (7.1-9.9)	**0.002**
CRP, mg/L	0.91 (0.58-1.42)	1.46 (1.10-1.95)	1.63 (1.15-2.31)	1.58 (1.27-1.96)	**0.03**
sICAM-1, ng/mL	250 (42)	296 (53)^a^	319 (59)^a^	307 (58)	**< 0.001**
sVCAM-1, ng/mL	594 (178)	602 (173)	662 (137)	629 (164)	0.36
s-eSelectin, ng/mL	47 (25)	63 (24)	66 (23)^a^	64 (23)	**0.002**
OxLDL/LDL	38 (31-45)	45 (40-51)	44 (38-49)	44	0.12
% current smokers	14	26	9	19	0.36
% on aspirin	3	4	16	9	0.33

Clinical and biochemical characteristics of healthy control subjects and Type 1 diabetic patients with microvascular complications (DMCx) and without complications (DMNoCx). Continuous data are mean (SD) or geometric mean (95% CI); ^a ^Significantly different from non-diabetic control subjects; ^b ^significantly different from Type 1 diabetic participants with microvascular complications.

Continuous data are mean (SD) or geometric mean (95% CI); ^a ^Significantly different from non-diabetic control subjects; ^b ^significantly different from Type 1 diabetic participants with microvascular complications.

### Increased kallistatin in diabetes and its complications

**Figure 1:**
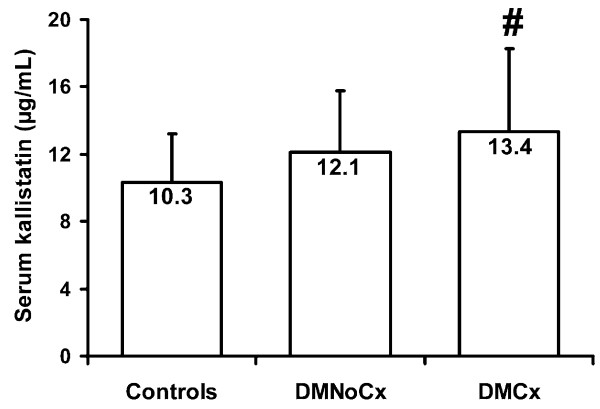
**Mean (SD) serum kallistatin levels in healthy non-diabetic control subjects, Type 1 diabetic patients without complications (DMNoCx) and Type 1 diabetic patients with complications (DMCx)**. Kallistatin levels differed between DMCx, DMNoCx, and controls, ANOVA, p = 0.007; # vs. control subjects p < 0.01.

Kallistatin levels mean(SD) were increased in the 116 diabetic vs. 29 controls (12.6(4.2) vs. 10.3(2.8) μg/ml; p = 0.007), and differed significantly between DMCx, DMNoCx, and controls (ANOVA, p = 0.007) (Figure 1). Levels were significantly higher in DMCx (13.4(4.9) μg/ml) than in control subjects, p = 0.01, but did not differ significantly between DMNoCx (12.1(3.7) μg/ml) and controls. Kallistatin levels were higher in DMCx vs. DMNoCx, but did not reach statistical significance (p = 0.25).

### Increased kallistatin concentrations in hypertension in diabetes and associations with poor vascular health

In diabetic patients, mean(SD) kallistatin levels were higher in those with (n = 54) vs. without (n = 62) diagnosed hypertension (13.5(4.7) vs. 11.8(3.5) μg/ml, p = 0.06). These groups differed significantly by age, diabetes duration, BMI, blood pressure, renal function, ESR and CRP, (not shown), but after adjustment for these covariates, adjusted mean (95%CI) kallistatin levels remained higher in those with vs. without hypertension (13.6(12.3-14.9) vs. 11.8(10.5-13.0) μg/ml, p = 0.03). In diabetes, kallistatin correlated inversely with SAE, r = -0.23, p = 0.02. In controls there were no statistically significant correlations with blood pressure or pulse-wave analysis indices. In the combined group, kallistatin correlated significantly with systolic and diastolic blood pressure and SAE (all p < 0.05).

### Kallistatin level correlate with renal dysfunction

On univariate analyses, in diabetes kallistatin concentrations correlated significantly with cystatin C, r = 0.28; p = 0.004, calculated GFR, r = -0.25; p = 0.009, urinary albumin/creatinine ratio, r = 0.34; p = 0.001, serum creatinine, r = 0.23; p = 0.01 and serum urea, r = 0.33, p = 0.001. There were no statistically significant (univariate analysis) correlations between kallistatin concentrations and renal function in the control group. In the combined groups kallistatin levels correlated with serum creatinine, urea, cystatin C, GFR and urinary albumin/creatinine ratio, (all p < 0.05).

### Kallistatin and lipid levels are correlated

In diabetes, kallistatin levels correlated with total cholesterol, r = 0.28, p = 0.004; LDL-cholesterol, r = 0.21, p = 0.03; and non-HDL-cholesterol, r = 0.21, p = 0.03; but not with triglycerides or HDL-cholesterol levels. In the control group, kallistatin levels correlated with total cholesterol, r = 0.39; p = 0.04, and in the combined groups with triglycerides, total-, LDL- and non-HDL-cholesterol (all p < 0.05). Kallistatin levels did not differ by lipid drug use in any group.

### Levels of Kallistatin, a hepatic product, and normal range liver function correlate

All liver function tests were within the normal range and levels of aminotransferases and bilirubin did not differ between diabetic and control groups. In both the diabetic and the combined diabetes and control groups, kallistatin levels correlated with gamma-glutamyltransferase (GGT) (r = 0.27, p = 0.04, and r = 0.25, p = 0.03 respectively), and inversely with bilirubin levels in the combined groups only, r = -0.22, p = 0.04.

### Kallistatin levels are not correlated with BMI, glycemia, inflammation or oxidative stress

In both diabetic and control groups, separately and combined, kallistatin levels did not differ by smoking status. In the diabetic group, but not in controls, kallistatin levels were significantly higher in females than in males: (13.3 (4.6) vs. 11.6 (3.4) μg/ml; p = 0.02). On univariate analyses, kallistatin did not correlate with BMI, measures of oxidative stress, or inflammation (not shown). In the combined groups kallistatin correlated with BMI, r = 0.19; p = 0.03, fasting blood glucose, r = 0.22; p = 0.01; and HbA1c, r = 0.26; p = 0.002, but these correlations were not statistically significant in the separate diabetes and control groups.

### Determinants of kallistatin levels

On forward stepwise linear regression analysis (all subjects), independent determinants of kallistatin were age, SAE, total cholesterol, fasting glucose, serum urea, and GGT (adjusted r^2 ^= 0.24, p < 0.00001), together accounting for 24% of variability in serum kallistatin levels. In diabetes, independent determinants of kallistatin were age, SAE, total cholesterol, serum urea and GGT (adjusted r^2 ^= 0.24, p < 0.00001). In controls: LAE, pulse pressure, total cholesterol, glucose, serum urea and WCC (adjusted r^2 ^= 0.49, p = 0.002). In each group and in the combined groups renal function (serum urea) was the strongest independent determinant (not shown).

## Discussion

In a cross-sectional study we identified increased serum kallistatin levels in Type 1 diabetic patients and in Type 1 diabetic patients with microvascular complications vs. age and gender-matched non-diabetic subjects. Further, kallistatin levels were elevated in diabetic subjects with hypertension vs. those without. There were no statistically significant differences in kallistatin levels between complication-free Type 1 diabetic patients and healthy subjects or between diabetic subjects with or without vascular complications. In the diabetic subjects considered as a single group, kallistatin levels were associated with renal dysfunction, total and LDL-cholesterol levels, and inversely with SAE, reflecting vascular dysfunction. Kallistatin levels in diabetes did not correlate with other lipids, glycemia, BMI, smoking, or measures of inflammation or oxidative stress. Common statistically independent determinants of kallistatin levels in all subjects and in the separate diabetes and control groups were renal function (most strongly) and cholesterol, with age, hepatic and vascular function also being related to kallistatin level variability in the combined group.

### Functions, sources, and levels of kallistatin

Kallistatin has vasodilatory, anti-angiogenic, anti-inflammatory and anti-oxidant effects [7-10,12,13]. It is localized in many human tissues, including eye, kidney, liver, heart, arteries and veins, atheroma, blood cells and body fluids [7,14,15,17]. The relative contributions of various cell and tissue types to circulating kallistatin levels in health and disease is unknown, but several studies support liver as a major source [7,14,25]. Hepatocytes secrete kallistatin [7,14,25] and in a small cross-sectional study of cirrhosis patients, circulating kallistatin levels were ≈30% that of healthy people [14]. In our study there were correlations between kallistatin levels and normal range liver function tests, perhaps in keeping with a predominant hepatic origin of serum kallistatin.

### Increased kallistatin in diabetes complications

To the best of our knowledge, the only prior study of kallistatin in human diabetes is by coauthor J-X Ma *et al *in which immunoreactive kallistatin levels in vitreous fluid from 18 patients with diabetic retinopathy were significantly lower compared to 17 non-diabetic subjects [17]. We now demonstrate higher serum kallistatin levels in Type 1 diabetic patients with vascular complications, which include proliferative retinopathy. We have noted a similar pattern with another serpin, Pigment Epithelium Derived Factor (PEDF), with low vitreous fluid levels in diabetic retinopathy patients and high serum levels with microvascular complications [23]. In the present study, kallistatin levels also related negatively to renal function, which could be due to reduced renal excretion or increased production, or both. Kallistatin has been localized in human urine [14,26] and in kidneys [7,14,27], where it is thought to regulate salt and water handling, renal perfusion and blood pressure, and to reduce intra-renal fibrosis, inflammation, and oxidative stress [7,12,14,28]. Apart from the current study, plasma, serum, renal tissue or urine kallistatin levels have not been reported in human diabetes or in other renal diseases. It may be that kallistatin levels rise in response to renal disease and proteinuria together with other circulating proteins of hepatic origin [29]. Urinary kallistatin excretion, not measured in this study as there were no urine kallistatin ELISA assays, merits future study.

### Kallistatin levels are associated with impaired vascular health

We observed positive associations between kallistatin and systolic blood pressure and pulse pressure, and an inverse correlation with SAE. Furthermore, serum kallistatin levels were higher in diabetic patients with vs. without diagnosed hypertension, even after statistical correction for renal dysfunction, and were also associated with impaired renal function by several measures. One possibility is that elevated kallistatin levels may be compensatory to mitigate the high blood pressure and endothelial dysfunction, as kallistatin is a potent vasodilator [7,10] and lowers blood pressure [30,31]. Other animal and isolated vascular cell experiments support a role of kallistatin in vascular biology, including vascular and cardiac remodeling [7,11] and angiogenesis [8,13]. Kallistatin increases *in vitro *growth, proliferation and migration of vascular smooth muscle cells and inhibits *in vitro *proliferation, migration, and adhesion of vascular endothelial cells [13]. In rats, balloon angioplasty markedly increased kallistatin mRNA and protein expression in injured vessels, which along with neointima formation was attenuated by local delivery of kallistatin antisense cDNA [13]. Kallistatin also inhibits angiogenesis in *in vivo *rat models of hind-limb ischemia and tumor growth [8].

### Kallistatin and inflammation and oxidative stress

The higher serum levels of inflammation markers (WCC, ESR and cell adhesion molecules) in diabetes and/or its complications in the present study are in keeping with other publications [20,32-34]. In our study, except for a positive correlation with WCC in controls on adjusted analyses, kallistatin levels were not significantly related to inflammation or oxidative stress measures. This contrasts with other literature [9,12,35,36]. In people with (inflammatory) rheumatoid arthritis, plasma and joint kallistatin levels were increased relative to osteoarthritis patients [35]. In animal studies, kallistatin gene delivery has anti-inflammatory and anti-oxidant effects, inhibiting renal inflammation including renal CAM expression in a rat renal disease model [12], inhibiting inflammation and apoptosis in acute myocardial ischemia-reperfusion injury [9], and reducing inflammation and joint injury in rat arthritis models [36]. Kallistatin levels decline during sepsis and severe inflammation, as markedly lower circulating kallistatin levels have been reported in humans with sepsis [14] and in necrotic acute pancreatitis [37]. In animal models hepatic kallistatin expression is reduced by lipopolysaccharide (LPS) [38], and transgenic mice overexpressing human kallistatin have lower LPS-induced mortality [39]. These previous studies support that kallistatin is an anti-inflammatory factor, but our present cross-sectional clinical study, in which serum kallistatin levels are not strongly associated with serum inflammation markers do not support a major anti-inflammatory role. This may relate to the level of inflammation in diabetes being relatively low, local tissue anti-inflammatory effects not being well-reflected by circulating measures, or to opposing effects of the effects of inflammation (decreasing kallistatin) and of renal and vascular dysfunction (increasing kallistatin).

### Angiogenesis and arteriogenesis in atherosclerosis

Neovascularisation, including angiogenesis and arteriogenesis (the rapid proliferation of pre-existing arterial vessels, which have a mature tunica media), is required to heal wounds and for collateral circulation development in ischemic tissues [40], common problems in diabetic patients [1,40]. However, angiogenesis within atheromatous plaques may be deleterious as leaky new vessel formation may promote inflammation, plaque growth, hemorrhage, instability, and rupture [40-43]. There is likely a delicate balance between pro- and anti-angiogenic factors, which likely varies at the different stages of blood vessel formation and repair and plaque formation, stability and regression. It is currently controversial as to whether pro- or anti-angiogenic factor based therapies will benefit atherosclerosis [44-46]. PEDF, another member of the serpin family, is now undergoing evaluation as a potential therapeutic agent for ocular angiogenesis [47]. The specific role and potential therapeutic effects of kallistatin in vascular disease, including atheroma progression and plaque stability, and specifically in the context of diabetes, remains unclear.

### Study limitations and future research

The limitations of a cross-sectional study are recognized, and kallistatin responses may vary by tissue in ways not necessarily reflected in serum levels. Longitudinal studies of kallistatin and the various types of diabetic complications are desirable. As higher kallistatin levels were inversely related to renal function, even in our study groups with relatively normal renal function, such as reflected by serum creatinine and urea, kallistatin levels in urine and in serum of people with different types and degrees of renal damage are merited. As we observed associations between kallistatin and blood pressure, vascular dysfunction, lipids and renal function, studies pre- and post-interventions targeting these factors are merited. Wound healing studies, mechanistic vascular reactivity studies involving diabetic animals, isolated vessels and plaque and cell culture models are relevant. Future studies may utilize additional measures of inflammation and oxidative stress.

## Conclusions

Increased serum kallistatin levels are associated with diabetic microvascular complications, hypertension and vascular dysfunction, renal dysfunction and elevated cholesterol, but not with measures of inflammation or oxidative stress. Whilst kallistatin's vasodilatory, potential anti-inflammatory and anti-oxidant effects may be beneficial; its potent anti-angiogenic effects may delay wound healing, impair collateral vessel formation and accelerate atherosclerosis.

## Competing interests

The authors declare that they have no competing interests.

## Authors' contributions

AJJ conceived of the study, designed it, recruited and characterized subjects, was involved in data analysis and wrote the manuscript. JMcB and BZ performed the kallistatin ELISAs and were involved in data analysis and writing the manuscript. ASJ, CSK, DNO, CLN and JSC, CAH participated in subject recruitment and characterization, including laboratory assays. CSK and ASJ also managed the database and ASJ performed the statistical analyses. TJL helped design the study and write the manuscript. J-XM conceived of and designed the study, was involved in the kallistatin ELISAs, data analysis and manuscript preparation. All authors read and approved the final manuscript.

## References

[B1] Jenkins AJ Best JD Klein RL Lyons TJ Lipoproteins, glycoxidation and diabetic angiopathy Diabetes Metab Res Rev 2004 20 349 368 10.1002/dmrr.491 15343582

[B2] Crawford TN Alfaro DV Kerrison JB Jablon EP Diabetic retinopathy and angiogenesis Curr Diabetes Rev 2009 5 1 8 13 10.2174/157339909787314149 19199892

[B3] Ma JX Zhang SX Wang JJ Down-regulation of angiogenic inhibitors: a potential pathogenic mechanism for diabetic complications Curr Diabetes Rev 2005 1 2 183 196 10.2174/1573399054022839 18220594

[B4] Chen S Ziyadeh FN Vascular endothelial growth factor and diabetic nephropathy Curr Diab Rep 2008 8 6 470 476 10.1007/s11892-008-0081-3 18990304

[B5] Moulton KS Angiogenesis in atherosclerosis: gathering evidence beyond speculation Curr Opin Lipidol 2006 17 5 548 555 10.1097/01.mol.0000245261.71129.f0 16960504

[B6] Chen CH Walterscheid JP Plaque angiogenesis versus compensatory arteriogenesis in atherosclerosis Circ Res 2006 99 8 787 789 10.1161/01.RES.0000247758.34085.a6 17038645

[B7] Chao J Miao RQ Chen V Chen LM Chao L Novel roles of kallistatin, a specific tissue kallikrein inhibitor, in vascular remodeling Biol Chem 2001 382 1 15 21 10.1515/BC.2001.003 11258665

[B8] Miao RQ Agata J Chao L Chao J Kallistatin is a new inhibitor of angiogenesis and tumor growth Blood 2002 100 9 3245 3252 10.1182/blood-2002-01-0185 12384424

[B9] Chao J Yin H Yao YY Shen B Smith RS Jr Chao L Novel role of kallistatin in protection against myocardial ischemia-reperfusion injury by preventing apoptosis and inflammation Hum Gene Ther 2006 17 12 1201 1213 10.1089/hum.2006.17.1201 17081080

[B10] Chao J Stallone JN Liang YM Chen LM Wang DZ Chao L Kallistatin is a potent new vasodilator J Clin Invest 1997 100 1 11 17 10.1172/JCI119502 9202051 PMC508159

[B11] Gao L Yin H S Smith R Jr Chao L Chao J Role of kallistatin in prevention of cardiac remodeling after chronic myocardial infarction Lab Invest 2008 88 11 1157 1166 10.1038/labinvest.2008.85 18762777

[B12] Shen B Hagiwara M Yao YY Chao L Chao J Salutary effect of kallistatin in salt-induced renal injury, inflammation, and fibrosis via antioxidative stress Hypertension 2008 51 5 1358 1365 10.1161/HYPERTENSIONAHA.107.108514 18391098

[B13] Miao RQ Murakami H Song Q Chao L Chao J Kallistatin stimulates vascular smooth muscle cell proliferation and migration in vitro and neointima formation in balloon-injured rat artery Circ Res 2000 86 4 418 424 10700446 10.1161/01.res.86.4.418

[B14] Chao J Schmaier A Chen LM Yang Z Chao L Kallistatin, a novel human tissue kallikrein inhibitor: levels in body fluids, blood cells, and tissues in health and disease J Lab Clin Med 1996 127 6 612 620 10.1016/S0022-2143(96)90152-3 8648266

[B15] Wolf WC Harley RA Sluce D Chao L Chao J Localization and expression of tissue kallikrein and kallistatin in human blood vessels J Histochem Cytochem 1999 47 2 221 228 9889257 10.1177/002215549904700210

[B16] Hatcher HC Ma JX Chao J Chao L Ottlecz A Kallikrein-binding protein levels are reduced in the retinas of streptozotocin-induced diabetic rats Invest Ophthalmol Vis Sci 1997 38 3 658 664 9071220

[B17] Ma JX King LP Yang Z Crouch RK Chao L Chao J Kallistatin in human ocular tissues: reduced levels in vitreous fluids from patients with diabetic retinopathy Curr Eye Res 1996 15 11 1117 1123 10.3109/02713689608995143 8950506

[B18] Hernández C Simó R Strategies for blocking angiogenesis in diabetic retinopathy: from basic science to clinical practice Expert Opin Investig Drugs 2007 16 8 1209 1226 10.1517/13543784.16.8.1209 17685870

[B19] Wilson AM O'Neal D Nelson CL Prior DL Best JD Jenkins AJ Comparison of arterial assessments in low and high vascular disease risk groups Am J Hypertens 2004 17 285 291 10.1016/j.amjhyper.2003.10.009 15062880

[B20] Nelson CL Karschimkus CS Dragicevic G Packham DK Wilson AM O'Neal D Becker GJ Best JD Jenkins AJ Systemic and vascular inflammation is elevated in early IgA and type 1 diabetic nephropathies and relates to vascular disease risk factors and renal function Nephrol Dial Transplant 2005 20 2420 2426 10.1093/ndt/gfi067 16115854

[B21] Lee AB Godfrey T Rowley KG Karschimkus CS Dragicevic G Romas E Clemens L Wilson AM Nikpour M Prior DL Best JD Jenkins AJ Traditional risk factor assessment does not capture the extent of cardiovascular risk in systemic lupus erythematosus Intern Med J 2006 36 4 237 243 10.1111/j.1445-5994.2006.01044.x 16640741

[B22] Wong M Toh L Wilson A Rowley K Karschimkus C Prior D Romas E Clemens L Dragicevic G Harianto H Wicks I McColl G Best J Jenkins A Reduced arterial elasticity in rheumatoid arthritis and the relationship to vascular disease risk factors and inflammation Arthritis Rheum 2003 48 1 81 89 10.1002/art.10748 12528107

[B23] Jenkins AJ Zhang SX Rowley KG Karschimkus CS Nelson CL Chung JS O'Neal DN Januszewski AS Croft KD Mori TA Dragicevic G Harper CA Best JD Lyons TJ Ma JX Increased serum pigment epithelium-derived factor is associated with microvascular complications, vascular stiffness and inflammation in Type 1 diabetes Diabet Med 2007 24 12 1345 1351 10.1111/j.1464-5491.2007.02281.x 17971181

[B24] Johnson CA Levey AS Coresh J Levin A Lau J Eknoyan G Clinical practice guidelines for chronic kidney disease in adults: Part II. Glomerular filtration rate, proteinuria, and other markers Am Fam Physician 2004 70 1091 1097 15456118

[B25] Chao J Chao L Biochemistry, regulation and potential function of kallistatin Biol Chem Hoppe Seyler 1995 376 12 705 713 9072045

[B26] Thongboonkerd V Malasit P Renal and urinary proteomics: current applications and challenges Proteomics 2005 5 4 1033 1042 10.1002/pmic.200401012 15669002

[B27] Chen LM Song Q Chao L Chao J Cellular localization of tissue kallikrein and kallistatin mRNAs in human kidney Kidney Int 1995 48 3 690 697 10.1038/ki.1995.339 7474653

[B28] Chao J Bledsoe G Yin H Chao L The tissue kallikrein-kinin system protects against cardiovascular and renal diseases and ischemic stroke independently of blood pressure reduction Biol Chem 2006 387 6 665 675 10.1515/BC.2006.085 16800727

[B29] Tessari P Kiwanuka E Barazzoni R Vettore M Zanetti M Diabetic nephropathy is associated with increased albumin and fibrinogen production in patients with type 2 diabetes Diabetologia 2006 49 8 1955 1961 10.1007/s00125-006-0288-2 16703327

[B30] Chen L-M Ma J-X Liang Y-M Chao L Chao J Tissue kallikrein-binding protein reduces blood pressure in transgenic mice J Biol Chem 1996 271 27590 27594 10.1074/jbc.271.44.27590 8910346

[B31] Chen L-M Chao L Chao J Adenoviral-mediated delivery of human kallistatin gene reduces blood pressure of spontaneously hypertensive rats Hum Gene Ther 1997 8 341 347 10.1089/hum.1997.8.3-341 9048201

[B32] Chen L-M Chao L Chao J Beneficial effects of kallikrein-binding protein in transgenic mice during endotoxic shock Life Sci 1996 60 1431 1436 10.1016/S0024-3205(97)00094-5 9126863

[B33] Jenkins AJ Rothen M Klein RL Moller K Eldridge L Zheng D Durazo-Arvizu R McGee D Lackland D Thorpe SR Garvey WT Lyons TJ DCCT/EDIC Research Group Cross-sectional associations of C-reactive protein with vascular risk factors and vascular complications in the DCCT/EDIC cohort J Diabetes Complications 2008 22 3 153 163 10.1016/j.jdiacomp.2007.02.003 18413218

[B34] Lopes-Virella MF Carter RE Gilbert GE Klein RL Jaffa M Jenkins AJ Lyons TJ Garvey WT Virella G Diabetes Control and Complications Trial/Epidemiology of Diabetes Intervention and Complications Cohort Study Group Risk factors related to inflammation and endothelial dysfunction in the DCCT/EDIC cohort and their relationship with nephropathy and macrovascular complications Diabetes Care 2008 31 10 2006 2012 10.2337/dc08-0659 18628568 PMC2551645

[B35] Wang CR Chen SY Shiau AL Wu CL Jou IM Chao L Chao J Upregulation of kallistatin expression in rheumatoid joints J Rheumatol 2007 34 11 2171 2176 17937475

[B36] Wang CR Chen SY Wu CL Liu MF Jin YT Chao L Chao J Prophylactic adenovirus-mediated human kallistatin gene therapy suppresses rat arthritis by inhibiting angiogenesis and inflammation Arthritis Rheum 2005 52 1319 1324 10.1002/art.20991 15818689

[B37] Bläckberg M Berling R Ohlsson K Tissue kallikrein in severe acute pancreatitis in patients treated with high-dose ntraperitoneal aprotinin Pancreas 1999 19 4 325 334 10.1097/00006676-199911000-00002 10547191

[B38] Chao J Chen LM Chai KX Chao L Expression of kallikrein-bonding protein and _1-antitrypsin genes in response to sex hormones, growth, inflammation and hypertension Agents Actions Suppl 1992 174 181 1466268 10.1007/978-3-0348-7321-5_23

[B39] Chen LM Chao L Chao J Beneficial effects of kallikrein-binding protein in transgenic mice during endotoxic shock Life Sci 1997 60 1431 1435 10.1016/S0024-3205(97)00094-5 9126863

[B40] Al Sabti H Therapeutic angiogenesis in cardiovascular disease J Cardiothorac Surg 2007 2 49 10.1186/1749-8090-2-49 18021404 PMC2169246

[B41] Di Stefano R Felice F Balbarini A Angiogenesis as risk factor for plaque vulnerability Curr Pharm Des 2009 15 10 1095 1106 10.2174/138161209787846892 19355951

[B42] Krupinski J Font A Luque A Turu M Slevin M Angiogenesis and inflammation in carotid atherosclerosis Front Biosci 2008 13 6472 6482 10.2741/3167 18508673

[B43] Ribatti D Levi-Schaffer F Kovanen PT Inflammatory angiogenesis in atherogenesis--a double-edged sword Ann Med 2008 40 8 606 621 10.1080/07853890802186913 18608127

[B44] Hoefer IE Timmers L Piek JJ Growth factor therapy in atherosclerotic disease-friend or foe Curr Pharm Des 2007 13 17 1803 1810 10.2174/138161207780831257 17584109

[B45] Esaki J Marui A Tabata Y Komeda M Controlled release systems of angiogenic growth factors for cardiovascular diseases Expert Opin Drug Deliv 2007 4 6 635 649 10.1517/17425247.4.6.635 17970666

[B46] Slevin M Kumar P Wang Q Kumar S Gaffney J Grau-Olivares M Krupinski J New VEGF antagonists as possible therapeutic agents in vascular disease Expert Opin Investig Drugs 2008 17 9 1301 1314 10.1517/13543784.17.9.1301 18694364

[B47] Tombran-Tink J PEDF in angiogenic eye diseases Curr Mol Med 2010 10 3 267 278 10.2174/156652410791065336 20236057

